# Supplementary material for: Sustained-input switches for transcription factors and microRNAs are central building blocks of eukaryotic gene circuits
Source: Genome Biol. 2013 Aug 23;14(8):R85. doi: 10.1186/gb-2013-14-8-r85 (PMC4054853; doi:10.1186/gb-2013-14-8-r85)
Supplement: Additional file 5 — HTML Browsable Motif Output. Zipped folder containing all WaRSwap and FANMOD motif output, viewable in a web browser. [file gb-2013-14-8-r85-S5.ZIP › HTML_browsable_motif_output/WaRSwap_ath_tair9/sigs_WSWRSLDraw_2-2000.pvals.heatmaps.html/motif_id_36_001011002_tftype_ath_upstream_-3000_0.html]

```
BG_MODEL = Rnew2
MOTIF_ID = 36_001011002
TF_TYPE = ath
UPSTREAM = -3000_0


PVals
FN_0.2	FN_0.4	FN_0.6	FN_0.8
dg_60.genes	0.014	0.051	0.642	1
dg_70.genes	0.013	0.05	0.62	1
dg_80.genes	0.009	0.048	0.518	1

ZScores
FN_0.2	FN_0.4	FN_0.6	FN_0.8
dg_60.genes	2.556	1.81	-0.413	-6.403
dg_70.genes	2.556	1.856	-0.295	-5.738
dg_80.genes	2.93	1.942	0.011	-4.848

StDevs
FN_0.2	FN_0.4	FN_0.6	FN_0.8
dg_60.genes	3552.882	2132.724	961.492	445.988
dg_70.genes	2592.67	1536.245	714.873	344.342
dg_80.genes	1058.525	697.668	343.613	194.677
```
